# Anxiety and depressive symptoms in college students during the late stage of the COVID-19 outbreak: a network approach

**DOI:** 10.1038/s41398-021-01738-4

**Published:** 2021-12-17

**Authors:** Wei Bai, Hong Cai, Shou Liu, Xu Chen, Sha Sha, Teris Cheung, Jessie Jingxia Lin, Xiling Cui, Chee H. Ng, Yu-Tao Xiang

**Affiliations:** 1 Unit of Psychiatry, Department of Public Health and Medicinal Administration, & Institute of Translational Medicine, Faculty of Health Sciences, University of Macau, Macao, SAR China; 2 Centre for Cognitive and Brain Sciences, University of Macau, Macao, SAR China; 3 Institute of Advanced Studies in Humanities and Social Sciences, University of Macau, Macao, SAR China; 4 Department of Public Health, Medical College, Qinghai University, Xining, Qinghai Province China; 5 The National Clinical Research Center for Mental Disorders & Beijing Key Laboratory of Mental Disorders, Beijing Anding Hospital & the Advanced Innovation Center for Human Brain Protection, Capital Medical University, Beijing, China; 6 School of Nursing, Hong Kong Polytechnic University, Hong Kong, SAR China; 7 Department of Rehabilitation Sciences, & Mental Health Research Centre, Hong Kong Polytechnic University, Hong Kong, SAR China; 8 Department of Business Administration, Hong Kong Shue Yan University, Hong Kong, China; 9 Department of Psychiatry, The Melbourne Clinic and St Vincent’s Hospital, University of Melbourne, Richmond, Victoria, Australia

**Keywords:** Diseases, Depression

## Abstract

Mental health problems are common in college students even in the late stage of the coronavirus disease 2019 (COVID-19) outbreak. Network analysis is a novel approach to explore interactions of mental disorders at the symptom level. The aim of this study was to elucidate characteristics of depressive and anxiety symptoms network in college students in the late stage of the COVID-19 outbreak. A total of 3062 college students were included. The seven-item Generalized Anxiety Disorder Scale (GAD-7) and nine-item Patient Health Questionnaire (PHQ-9) were used to measure anxiety and depressive symptoms, respectively. Central symptoms and bridge symptoms were identified based on centrality and bridge centrality indices, respectively. Network stability was examined using the case-dropping procedure. The strongest direct relation was between anxiety symptoms “Nervousness” and “Uncontrollable worry”. “Fatigue” has the highest node strength in the anxiety and depression network, followed by “Excessive worry”, “Trouble relaxing”, and “Uncontrollable worry”. “Motor” showed the highest bridge strength, followed by “Feeling afraid” and “Restlessness”. The whole network was robust in both stability and accuracy tests. Central symptoms “Fatigue”, “Excessive worry”, “Trouble relaxing” and “Uncontrollable worry”, and critical bridge symptoms “Motor”, “Feeling afraid” and “Restlessness” were highlighted in this study. Targeting interventions to these symptoms may be important to effectively alleviate the overall level of anxiety and depressive symptoms in college students.

## Introduction

Depressive and anxiety symptoms (depression and anxiety hereafter) are common mental health problems, which are increasing globally in the past decade [1]. The presence of either depression or anxiety often increases the risk of having the other. For instance, a meta-analysis revealed that depression and anxiety are bidirectional risk factors for one another [2]. In addition, depression and anxiety often occur concurrently, such as in a study on UK college students, 29.8% of females and 13.9% of males screened positive for both anxiety and depression [3].

The coronavirus disease 2019 (COVID-19) outbreak that started in early 2020 have resulted in an increase in common depression and anxiety across many populations [4–6]. After the COVID-19 outbreak was largely controlled in some countries such as China, studies found that the large scale public health measures (e.g., quarantine, self-isolation, and business and school closures) resulted in long-term stress and psychological distress in many populations [7, 8] such as college students [9]. Compared to most other subpopulations, college students are more likely to experience mental health problems [10]. The COVID-19 resurgence caused by imported cases and relevant public health measures often lead to depression and anxiety in students due to fear of immediate quarantine, delays in school opening, and switching to online teaching [11]. A recent two-wave longitudinal survey in China found that the rates of depression and anxiety among college students increased in the late stage of the COVID-19 outbreak compared to that in the early stage [12], indicating that long-term preventive measures and mental health services are important for this population even in the post-outbreak period.

In the last few years, the network analysis has been widely used in psychopathology to conceptualize and visualize patterns relevant to psychiatric disorders. In the theory of network analysis, psychiatric disorders consist of interacting symptoms [13, 14]; accurate descriptions of these interactions are crucial to explain potential psychopathological mechanisms and develop effectively targeted intervention strategies [15]. In the visualization of the network model, each symptom of a psychiatric disorder can be viewed as a node and the association between two symptoms is viewed as an edge [16]. Compared with the traditional method of using total scale scores, network analysis is a symptom-oriented approach which can calculate indices for each node, such as centrality and predictability, representing a node’s importance and controllability in a network [17, 18]. Calculating centrality indices could be beneficial to identify central (influential) symptoms in a psychiatric disorder, and these symptoms may be potential targets to prevention and interventions. Additionally, this novel model is useful in understanding comorbidities [19]. When an individual suffers a particular psychiatric disorder, the symptoms of this disorder may increase the risk of other disorders, which is regarded as bridge symptom in network model. The bridge symptoms in the network play an important role in maintaining and developing comorbidities, and provide hints for clinicians to prevent and treat comorbidities [19].

Researchers have explored characteristics of the anxiety and depression network in various populations. For example, “fatigue” was identified as the central and bridge symptom in migrant Filipino domestic workers, which may increase the risk of comorbidity between anxiety and depression [20]. In another psychiatric sample, “sad mood” and “worry” were the two most central symptoms in the network [21], suggesting that targeting these symptoms in treatment would be more effective. Convincing evidence has shown that patterns and features of mood disorders were influenced by socioeconomic contexts [22, 23], suggesting that the network structure of anxiety and depressive symptoms should be examined separately across populations of different socioeconomic backgrounds.

To date, no studies have investigated how depressive and anxiety symptoms are related to each other in college students using the network model, particularly in the late stage of the COVID-19 outbreak, which gives us the impetus to conduct this study. The aim of the present study was hence to examine the associations between depressive and anxiety symptoms in Chinese college students in the late stage of the COVID-19 outbreak using network analysis.

## Methods

### Study settings and participants

This was a nationwide survey conducted among Chinese college students between December 27, 2020 and March 12, 2021, which was considered the late stage of the COVID-19 outbreak in China. To avoid the risk of face-to face transmission, online questionnaires were distributed using snowball sampling. The details of survey procedures have been introduced elsewhere [24]. To be eligible, participants need to be undergraduate students aged between 16 and 30 years, Chinese ethnicity, and able to understand the purpose and content of this survey. This study was approved by the Institutional Review Board (IRB) of Beijing Anding Hospital. All participants have provided electronic written informed consent; guardians provided informed consent if students were younger than 18 years.

### Measures

The nine-item Patient Health Questionnaire (PHQ-9) was used to assess depressive symptoms [25]. Reference names of each item in the network analysis are presented in Table 1. The PHQ-9 is a four-point Likert scale with each item scored from 0 (not at all) to 3 (nearly every day); higher scores indicates more severe symptoms. The Chinese version of PHQ-9 was used as it has been well validated in Chinese populations [26].

**Table 1:** Descriptive statistics of the PHQ-9 and GAD-7 items.

Item abbreviations	Item content	Item mean (SD)	Node strength^a^	Predictability
GAD1	Nervousness	0.64 (0.82)	0.921	0.521
GAD2	Uncontrollable worry	0.55 (0.81)	1.042	0.675
GAD3	Excessive worry	0.68 (0.86)	1.063	0.688
GAD4	Trouble relaxing	0.63 (0.86)	1.053	0.667
GAD5	Restlessness	0.42 (0.74)	0.996	0.632
GAD6	Irritability	0.60 (0.82)	1.033	0.648
GAD7	Feeling afraid	0.42 (0.74)	0.910	0.589
PHQ1	Anhedonia	0.81 (0.85)	0.801	0.484
PHQ2	Sad Mood	0.71 (0.78)	1.007	0.583
PHQ3	Sleep	0.71 (0.90)	0.739	0.436
PHQ4	Fatigue	0.80 (0.84)	1.096	0.608
PHQ5	Appetite	0.60 (0.83)	0.808	0.460
PHQ6	Guilty	0.66 (0.86)	0.944	0.536
PHQ7	Concentration	0.63 (0.84)	0.929	0.544
PHQ8	Motor	0.39 (0.72)	0.991	0.562
PHQ9	Suicide	0.21 (0.56)	0.623	0.380

*GAD-7* 7-item Generalized Anxiety Disorder Scale, *PHQ-9* the 9-item Patient Health Questionnaire, *SD* standard deviation

^a^The values of node strength were raw data from the network

Anxiety symptoms were measured using the seven-item Generalized Anxiety Disorder (GAD-7) [27] scale and the reference names of items are shown in Table 1. This is also a four-point Likert scale, with each item scored from 0 (not at all) to 3 (nearly every day). Higher scores indicate more severe anxiety symptoms. The Chinese version of the GAD-7 has satisfactory psychometric properties [28].

### Data analysis

All analyses were conducted using the R program [29]. The network analysis was performed in three domains, including network estimation, network stability, and network comparisons.

#### Network estimation

In the parlance of network analysis, each item is indicated as a node and the association between two nodes is viewed as an edge. The association between each pairwise nodes was computed with partial correlation analysis, controlling for the confounding effects of all the other nodes. The least absolute shrinkage and selection operator (LASSO) was used to shrink all edges in the network and set small correlations to zero [30], which enables nodes with as few edges as necessary to be retained in the network. The extended Bayesian Information Criteria (EBIC) was adopted to choose related turning parameter so that the network was sparser and easier to interpret [31]. Due to the skewed distribution of the mean item scores, nonparametric correlations were calculated by the nonparanormal transformation [32]. The R packages *bootnet* (Version 1.4.3) [33] and *qgraph* (Version 1.6.9) [16] were used to estimate and visualize the network. In the layout of network, the thickness of edges indicates the magnitude of the association. Blue edges referred to positive associations, while red edges indicated negative ones.

To quantify the importance of each node in the network, centrality indices were computed using the function *centralityPlot* of the R package *qgraph* (Version 1.6.9) [16]. The network was usually characterized with the several centrality indices, including strength, betweenness, and closeness [34]. Previous studies demonstrated that estimations of closeness and betweenness are unreliable [35, 36], thus, the most often used centrality index of strength was used in this study. Predictability was also measured in this study, which indicates the interconnectedness and the extent of a node associated with its neighboring nodes [31]. In the layout of the network, the area in the rings around each node represents the value of predictability, which was calculated using the function *predict* of R package *mgm* (Version 1.2-11) [37].

To assess the importance of a node in linking anxiety and depression, as recommended in previous studies [19], bridge centrality index of bridge strength was analyzed using the function *bridge* of the R package *networktools* (Version 1.2.3) [38].

#### Network stability

The stability of node strength and bridge strength was examined using a case-dropping bootstrap procedure. In this procedure, a growing percentage of cases was dropped from the dataset, while the centrality indices were re-estimated. A network is stable if a large proportion of sample could be excluded from dataset without observing significant changes of indices, and the stability is quantified by the Correlation Stability Coefficient (CS-C) [33]. The CS-C means the maximum cases that could be dropped from the sample, in which the centrality indices from the subsamples are correlated with the indices from the original sample at a value of *r* = 0.7 [33]. Generally, the value of CS-C needs to be above 0.25 and is preferably above 0.5 [33]. A nonparametric bootstrap procedure was used to assess the edge weights stability based on the 95% confidence intervals (95% CIs). Edge accuracy was assessed by 95% CIs, with a narrower CI indicating a more trustworthy network [33, 39]. Additionally, to evaluate the differences between two edges or between two nodes strength, bootstrapped tests were conducted based on 95% CIs, which indicated that there were statistical differences between two edges or two nodes strength if zero was not included in the CIs [33]. All analyses in network stability were performed by the R package *bootnet* (Version 1.4.3) [33].

#### Network comparison

The Network Comparison Test (NCT) in the R-package *NetworkComparisonTest* (Version 2.2.1) was used to examine the three invariance measures (i.e., network structure invariance, edge invariance, and global strength) [40]. Network structure means the maximum difference of pairwise edges between two networks, edge invariance indicates the difference of individual edge weight between two networks, and global strength refers to the sum of all edges of each network. Holm-Bonferroni correction for multiple comparisons at the level of individual edge between two networks was adopted. Considering the moderating effect of gender [41], academic major [42] and living area [42, 43] on anxiety and depression among college students, network structure invariance, edge invariance, and global strength were compared between different subgroups (e.g., between females and males, between health-related major and others, and between rural and urban residents) based on a permutation test (*n* = 1000) [40].

## Results

### Descriptive statistics

Out of the 3075 college students invited to participate, 3,062 agreed and completed the assessment, giving a response rate of 99.58%. Of the 3,062 college students included in this network, the mean age was 19.8 (standard deviation (SD) = 2.0) years, 2,068 (67.5%) were females, 1563 (51.0%) were rural residents, and 1722 (56.2%) majored in health-related subjects (Table S1). The mean PHQ-9 and GAD-7 rating score was 0.21 and 0.80, respectively (Table 1), and the distributions of the responses to PHQ-9/GAD-7 items are shown in Table S2.

### Network structure

**Fig. 1:**
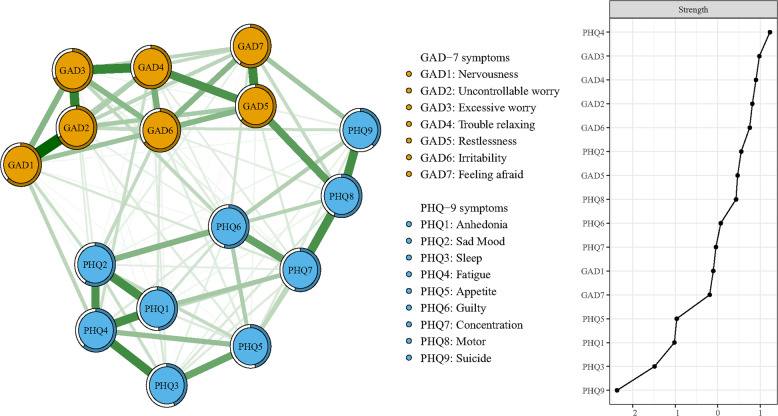
Network structure of anxiety and depressive symptoms in college students. The left panel shows the visualization of the network structure; the right panel shows the value of strength in order.

The network of anxiety and depressive symptoms is shown in Fig. 1 and the corresponding partial correlation matric is presented in Table S3. The edge Nervousness-Uncontrollable worry (GAD1-GAD2) shows the strongest association, followed by the edge Uncontrollable worry-Excessive worry (GAD2-GAD3), Excessive worry-Trouble relaxing (GAD3-GAD4), Restless-Feeling afraid (GAD5-GAD7), Sleep-Fatigue (PHQ3-PHQ4), Motor-Suicide (PHQ8-PHQ9), Anhedonia-Sad Mood (PHQ1-PHQ2), and Concentration-Motor (PHQ7-PHQ8).

**Fig. 2:**
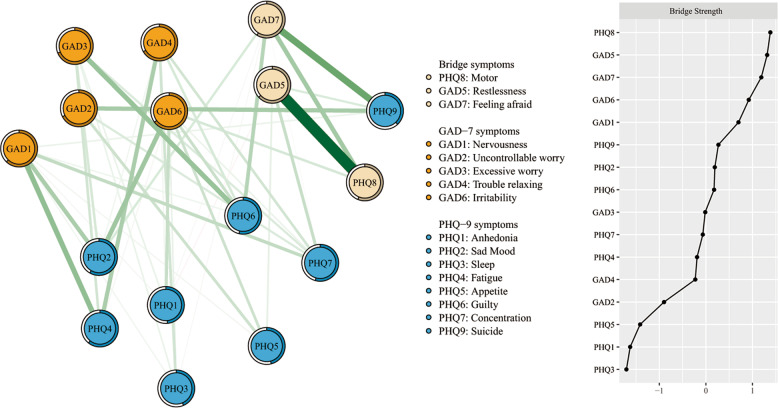
Network structure of anxiety and depressive symptoms showing bridge symptoms in college students. The left panel shows the visualization of the network structure of bridging symptoms; the right panel shows the value of bridge strength in order.

In Table 1 and Fig. 1, Fatigue (PHQ4) has the highest node strength in the anxiety and depression network among college students, followed by Excessive worry (GAD3), Trouble relaxing (GAD4), and Uncontrollable worry (GAD2). The item Excessive worry (GAD3) had the highest predictability in the network (Table 1) and an average of 56.3% of variance could be potentially accounted for by each node’s surrounding nodes (*M*_predictability_ = 0.563 ± 0.091). In terms of bridge symptoms, Motor (PHQ8) showed the highest bridge strength, followed by Feeling afraid (GAD7) and Restlessness (GAD5) (Fig. 2).

### Network stability

**Fig. 3:**
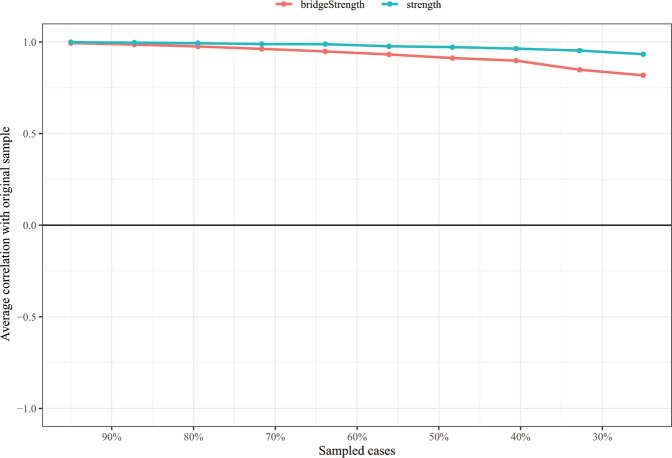
The stability of strength and bridge strength using case-dropping bootstrap. The x-axis indicates the percentage of cases of the original sample included at each step. The y-axis indicates the average of correlations between the centrality indices from the original network and the centrality indices from the networks that were re-estimated after excluding increasing percentages of cases.

In Fig. 3, the case-dropping bootstrap procedure shows that both CS-Cs of node strength and bridge strength were 0.75, which indicates that 75% of samples could be dropped, but the findings were still similar to the primary results (*r* = 0.7). The results of nonparametric bootstrap procedure show that most comparisons among edge weights and node strength were statistically significant (Figs. S1, S2). Additionally, bootstrapped 95% CIs were narrow, representing edges were trustworthy (Fig. S3).

### Network comparisons

As shown in Fig. S4, there was significant difference in network global strength (Urban: 7.655 vs Rural: 7.469, *S* = 0.186, *p* = 0.044) between rural and urban college students. In other two subsample comparisons, no significant differences were found in network global strength (Health-related major: 7.456 vs Other majors: 7.431, *S* = 0.025, *p* = 0.703; Females: 7.461 vs Males: 7.496, *S* = 0.035, *p* = 0.613). In terms of network structure and individual edge weight comparisons, there were also no significant differences between two networks in the three subsample comparisons.

## Discussion

To the best of our knowledge, this was the first study that characterized the depressive and anxiety network in Chinese college students during the late stage of the COVID-19 outbreak. All the strongest edges were within the respective disorder, while none of the strongest edges linked anxiety and depressive symptoms, which are consistent with previous findings identified in network analysis of depression and anxiety [15, 20, 21].

In the whole depression and anxiety network, all the top four strongest edges existed in the anxiety community, which are different from previous studies [15, 20] where edges between depressive symptoms were the strongest in depression and anxiety network. This discrepancy may be due to different study samples; i.e., college students in this study vs. domestic workers in the Garabiles et al.’s study [20] and nursing students in the Ren et al.’s study [15]. The strongest edge in the whole depression and anxiety network was the connection between the “Nervousness” (GAD1) and “Uncontrollable worry” (GAD2), which could be due to the following reasons. Due to occasional small-scale outbreaks caused by imported cases from overseas, feelings of helplessness, fear, and apprehension were common stressors among college students [44]. Furthermore, classroom teaching may be interrupted at any time due to resurgence caused by imported cases from overseas. In switching to online learning, many college students are unable to adjust to online teaching [44], which may increase anxiety about the academic burden [45]. Apart from the influential edges within anxiety symptoms, several strong edges within depressive symptoms were observed. The edge between “Sleep” (PHQ3) and "Fatigue (PHQ4) is the strongest one, which was also the strongest edge in the depression and anxiety network study among nursing students [15]. This could be explained by the sudden change to sedentary lifestyle with reduced outdoor physical activities due to public health measures, resulting in increased fatigue [46]. Additionally, although sleep quantity could increase among the population during the lockdown, the sleep quality was often poorer [46, 47], which could increase the risk of fatigue. Based on our findings, specific interventions that improve sleep quality and increase physical exercise may be helpful to alleviate depressive and anxiety problems.

Node index of strength may be crucial in identifying influential symptoms that activate and maintain psychopathological networks, and that are potential targets of interventions [15, 39]. “Fatigue” (PHQ4) had the highest strength in the whole network, indicating its important role in the network of depression and anxiety. This is consistent with previous findings in nursing students [15], Filipino domestic workers [20], and patients with major depressive disorders [48]. Fatigue is common in students in the late stage of the COVID-19 outbreak, and a recent study found that the prevalence of fatigue was 67.3% (95% CI: 64.4–70.0%) in nursing students in this period [46]. Recent studies also found that fatigue in college students during the COVID-19 outbreak may be related to several factors including increased academic burden, inadequate physical activities, and poor sleep quality [15, 44, 46, 47]. Moreover, compared with verbal expression, physical expression is often used as a coping strategy in Chinese societies, which may be associated with fatigue [15]. In this study, we found that certain anxiety symptoms, including “Excessive worry” (GAD3), “Trouble relaxing” (GAD4), and “Uncontrollable worry” (GAD2), also had high values of node strength, indicating these symptoms may also play important role in activating and maintaining the depression and anxiety network. This could be partly explained by the fear of contagion when students are faced with this novel and potentially fatal infectious disease, which can increase such anxiety symptoms [44]. Specific interventions could be adopted, such as cognitive behavioral therapy (CBT), applied relaxation and medications, the latter being considered for those with severe symptoms.

In this depression and anxiety network, the most influential bridge symptom was the depressive symptom of “Motor” (PHQ8), which is similar to that in a previous study in Chinese adults, where “Motor” (PHQ8) showed a high bridge centrality both during the COVID-19 peak and post-peak outbreak period [49]. In another study, the symptom of “Motor” was identified as the crucial priority due to its relation to “thought of death” in female nursing students [15], suggesting that this symptom should be a target of interventions to reduce depression and anxiety. Other influential bridge symptoms included the anxiety symptoms of “Feeling afraid” (GAD7) and “Restlessness” (GAD5), suggesting that these symptoms should also be targeted in treatment.

The predictability of each node in the network of depression and anxiety was calculated. There were no associations between predictability and mean values of each node (*r*_*s*_ = −0.056, *p* = 0.837), suggesting that certain symptoms might have a high value of predictability in the depressive and anxiety network, although these symptoms appeared less frequently [39]. On average, 56.3% of the node variance could be explained by neighboring nodes, implying that the potential sources of the remaining variance (e.g., stress and insomnia symptoms) were not included by both the PHQ-9 and GAD-7. Previous studies found that certain factors, such as gender, living area (urban/rural), and study major, were associated with depression and anxiety at the disorder level [41–43]. In this study, network comparison test found that compared to those from rural areas, students from urban areas had a significantly higher global strength of the network, indicating that individual symptoms in the model of urban college students were strongly inter-connected. This finding was not found in the relevant studies using network analysis and should be explored in future studies. In other comparisons (such as health-related major vs. other majors, and female vs male), no significant differences were found.

The strength of this study included the large sample size and use of the network approach to visualize depressive and anxiety symptom patterns in college students, with stable results. However, several limitations should be noted. First, the cross-sectional data collected by snowball sampling method were used to construct depressive and anxiety symptoms network structure, which could not identify the causality between individual symptoms and had limited representativeness. Therefore, the findings should be confirmed in future longitudinal studies. Second, self-reported measures were used to assess depressive and anxiety symptoms, which may have recall bias and are limited to capture clinical phenomena [15]. Third, for logistical reasons, depressive and anxiety network prior to and in the early stage of the COVID-19 pandemic were not assessed. Hence, the psychological impact of the pandemic could not be evaluated. Finally, some relevant symptoms, such as post-traumatic stress and certain somatic symptoms, were not measured, which could partly explain the relatively low predictability in the network.

In conclusion, centrality symptoms (i.e., “Fatigue”, “Excessive worry”, “Trouble relaxing” and “Uncontrollable worry”) and bridge symptoms (i.e., “Motor”, “Feeling afraid” and “Restlessness”) were identified in this network of depressive and anxiety symptoms in Chinese college students. Monitoring college students’ mental health in the late stage of the COVID-19 outbreak and targeting interventions (e.g., CBT, applied relaxation and medications) for selective symptoms are important to alleviate the overall level of anxiety and depression in this population.

## Supplementary information

Supplementary material
