# Supplementary material for: Anxiety and depressive symptoms in college students during the late stage of the COVID-19 outbreak: a network approach
Source: Transl Psychiatry. 2021 Dec 17;11:638. doi: 10.1038/s41398-021-01738-4 (PMC8678580; doi:10.1038/s41398-021-01738-4)
Supplement: Supplementary file 1 — Supplementary material [file 41398_2021_1738_MOESM1_ESM.docx]

**Supplementary materials**

Table S1. Summary of participants characteristics

Table S2. The distributions of the responses to PHQ-9 and GAD-7 items

Table S3. Correlation matrix of the PHQ-9 and GAD-7 items

Figure S1. Bootstrapped stability test for ‘node strength’

Figure S2. Bootstrapped stability test for edge-weight

Figure S3. Bootstrapped confidence intervals of all edges

Figure S4. Network comparisons of anxiety and depressive symptoms by majors, genders and living areas in college students

Table S1. Summary of participant characteristics

| Variables | Mean (SD) or N (%) |
| --- | --- |
| Age (years) | 19.8 (2.0) |
| Female gender | 2,068 (67.5) |
| Rural | 1,563 (51.0) |
| Health-related major | 1,722 (56.2) |
| Academic grade |  |
| First year | 1,732 (56.6) |
| Second year | 642 (21.0) |
| Third year | 342 (11.2) |
| Fourth year | 242 (7.9) |
| Fifth year | 104 (3.4) |
| Note: SD, standard deviation. | |

Table S2. The distributions of the responses to PHQ-9 and GAD-7 items

| Items | Answers, n(%) | | | |
| --- | --- | --- | --- | --- |
|  | Not at all | Several days | More than half days | Nearly every day |
| PHQ-9 |  |  |  |  |
| PHQ1 | 1,291 (42.2) | 1,214 (39.6) | 407 (13.3) | 150 (4.9) |
| PHQ2 | 1,422 (46.4) | 1,193 (39.0) | 357 (11.7) | 90 (2.9) |
| PHQ3 | 1,612 (52.6) | 915 (29.9) | 348 (11.4) | 187 (6.1) |
| PHQ4 | 1,286 (42.0) | 1,241 (40.5) | 389 (12.7) | 146 (4.8) |
| PHQ5 | 1,791 (58.5) | 839 (27.4) | 310 (10.1) | 122 (4.0) |
| PHQ6 | 1,688 (55.1) | 879 (28.7) | 350 (11.4) | 145 (4.7) |
| PHQ7 | 1,732 (56.6) | 865 (28.2) | 342 (11.2) | 123 (4.0) |
| PHQ8 | 2,234 (73.0) | 529 (17.3) | 235 (7.6) | 64 (2.1) |
| PHQ9 | 2,609 (5.2) | 292 (9.5) | 131 (4.3) | 30 (1.0) |
| GAD-7 |  |  |  |  |
| GAD1 | 1,651 (53.9) | 984 (32.1) | 306 (10.0) | 121 (4.0) |
| GAD2 | 1,876 (61.3) | 793 (25.9) | 273 (8.9) | 120 (3.9) |
| GAD3 | 1,619 (52.9) | 960 (31.3) | 328 (10.7) | 155 (5.1) |
| GAD4 | 1,756 (57.3) | 829 (27.1) | 325 (10.6) | 152 (5.0) |
| GAD5 | 2,153 (70.3) | 617 (20.2) | 211 (6.9) | 81 (2.6) |
| GAD6 | 1,764 (57.6) | 879 (28.7) | 303 (9.9) | 116 (3.8) |
| GAD7 | 2,159 (70.5) | 602 (19.7) | 220 (7.2) | 81 (2.6) |
| Note: GAD, Generalized Anxiety Disorder; PHQ, Patient Health Questionnaire. | | | | |

Table S3. Correlation matrix of the PHQ-9 and GAD-7 items

|  | PHQ1 | PHQ2 | PHQ3 | PHQ4 | PHQ5 | PHQ6 | PHQ7 | PHQ8 | PHQ9 | GAD1 | GAD2 | GAD3 | GAD4 | GAD5 | GAD6 | GAD7 |
| --- | --- | --- | --- | --- | --- | --- | --- | --- | --- | --- | --- | --- | --- | --- | --- | --- |
| PHQ1 |  | 0.2185 | 0.0472 | 0.2194 | 0.0408 | 0.0573 | 0.1065 | 0.0190 | 0.0000 | 0.0082 | 0.0000 | 0.0163 | 0.0101 | -0.0083 | 0.0428 | 0.0062 |
| PHQ2 | 0.2185 |  | 0.0640 | 0.2115 | 0.0701 | 0.1451 | 0.0341 | 0.0260 | 0.0225 | 0.0639 | 0.0058 | 0.0320 | 0.0118 | 0.0000 | 0.0715 | 0.0303 |
| PHQ3 | 0.0472 | 0.0640 |  | 0.2252 | 0.1769 | 0.0310 | 0.0387 | 0.0440 | 0.0256 | 0.0113 | 0.0000 | 0.0156 | 0.0359 | 0.0000 | 0.0167 | 0.0071 |
| PHQ4 | 0.2194 | 0.2115 | 0.2252 |  | 0.1251 | 0.0404 | 0.0760 | 0.0076 | 0.0013 | 0.0806 | 0.0026 | 0.0340 | 0.0637 | 0.0000 | 0.0085 | 0.0000 |
| PHQ5 | 0.0408 | 0.0701 | 0.1769 | 0.1251 |  | 0.0965 | 0.0758 | 0.0748 | 0.0413 | 0.0105 | 0.0389 | 0.0000 | 0.0000 | 0.0196 | 0.0375 | 0.0000 |
| PHQ6 | 0.0573 | 0.1451 | 0.0310 | 0.0404 | 0.0965 |  | 0.1839 | 0.0854 | 0.0903 | 0.0130 | 0.0000 | 0.0762 | 0.0308 | 0.0000 | 0.0394 | 0.0548 |
| PHQ7 | 0.1065 | 0.0341 | 0.0387 | 0.0760 | 0.0758 | 0.1839 |  | 0.2164 | 0.0000 | 0.0471 | 0.0272 | 0.0271 | 0.0344 | 0.0420 | 0.0154 | 0.0045 |
| PHQ8 | 0.0190 | 0.0260 | 0.0440 | 0.0076 | 0.0748 | 0.0854 | 0.2164 |  | 0.2219 | 0.0000 | 0.0006 | 0.0000 | 0.0000 | 0.1937 | 0.0329 | 0.0688 |
| PHQ9 | 0.0000 | 0.0225 | 0.0256 | 0.0013 | 0.0413 | 0.0903 | 0.0000 | 0.2219 |  | 0.0152 | 0.0663 | 0.0000 | 0.0000 | 0.0278 | 0.0000 | 0.1111 |
| GAD1 | 0.0082 | 0.0639 | 0.0113 | 0.0806 | 0.0105 | 0.0130 | 0.0471 | 0.0000 | 0.0152 |  | 0.3037 | 0.1425 | 0.0872 | 0.0000 | 0.1160 | 0.0218 |
| GAD2 | 0.0000 | 0.0058 | 0.0000 | 0.0026 | 0.0389 | 0.0000 | 0.0272 | 0.0006 | 0.0663 | 0.3037 |  | 0.2436 | 0.0969 | 0.1040 | 0.0716 | 0.0808 |
| GAD3 | 0.0163 | 0.0320 | 0.0156 | 0.0340 | 0.0000 | 0.0762 | 0.0271 | 0.0000 | 0.0000 | 0.1425 | 0.2436 |  | 0.2382 | 0.0164 | 0.1400 | 0.0815 |
| GAD4 | 0.0101 | 0.0118 | 0.0359 | 0.0637 | 0.0000 | 0.0308 | 0.0344 | 0.0000 | 0.0000 | 0.0872 | 0.0969 | 0.2382 |  | 0.2125 | 0.1589 | 0.0723 |
| GAD5 | -0.0083 | 0.0000 | 0.0000 | 0.0000 | 0.0196 | 0.0000 | 0.0420 | 0.1937 | 0.0278 | 0.0000 | 0.1040 | 0.0164 | 0.2125 |  | 0.1417 | 0.2300 |
| GAD6 | 0.0428 | 0.0715 | 0.0167 | 0.0085 | 0.0375 | 0.0394 | 0.0154 | 0.0329 | 0.0000 | 0.1160 | 0.0716 | 0.1400 | 0.1589 | 0.1417 |  | 0.1404 |
| GAD7 | 0.0062 | 0.0303 | 0.0071 | 0.0000 | 0.0000 | 0.0548 | 0.0045 | 0.0688 | 0.1111 | 0.0218 | 0.0808 | 0.0815 | 0.0723 | 0.2300 | 0.1404 |  |
| Note: GAD, Generalized Anxiety Disorder; PHQ, Patient Health Questionnaire; PHQ1: Anhedonia, PHQ2: Sad Mood, PHQ3: Sleep, PHQ4: Fatigue, PHQ5: Appetite, PHQ6: Guilty, PHQ7: Concentration, PHQ8: Motor, PHQ9: Suicide; GAD1: Nervousness, GAD2: Uncontrollable worry, GAD3: Excessive worry, GAD4: Trouble relaxing, GAD5: Restless, GAD6: Irritability, GAD7: Feeling afraid. | | | | | | | | | | | | | | | | |


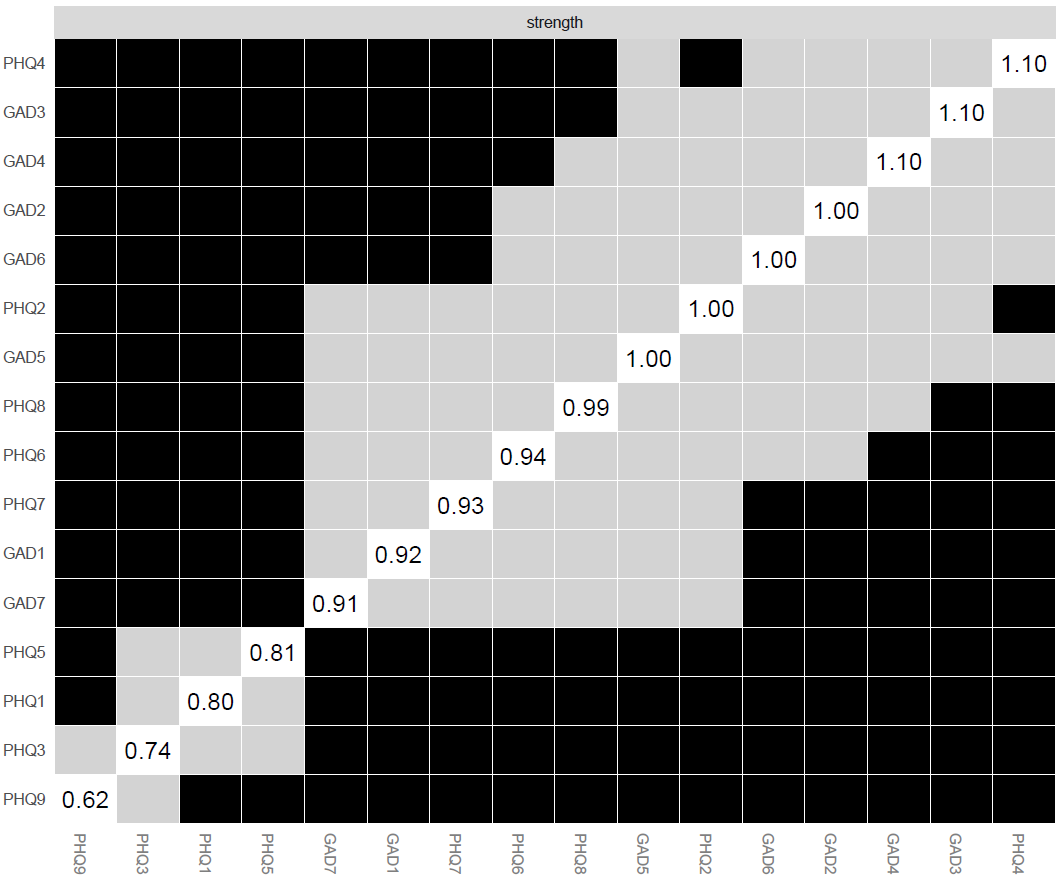


Figure S1. Bootstrapped stability test for ‘node strength’

PHQ1: Anhedonia, PHQ2: Sad Mood, PHQ3: Sleep, PHQ4: Fatigue, PHQ5: Appetite, PHQ6: Guilty, PHQ7: Concentration, PHQ8: Motor, PHQ9: Suicide; GAD1: Nervousness, GAD2: Uncontrollable worry, GAD3: Excessive worry, GAD4: Trouble relaxing, GAD5: Restlessness, GAD6: Irritability, GAD7: Feeling afraid; The stability difference tests (α=0.05) for ‘node strength’ are shown in this Figure. The color of the boxes indicates whether there is a significant difference between symptoms (i.e., grey boxes reflect no significant differences and black boxes reflect significant differences). The number in the white boxes (i.e., the diagonal line) denotes the value of node strength of a specific node.


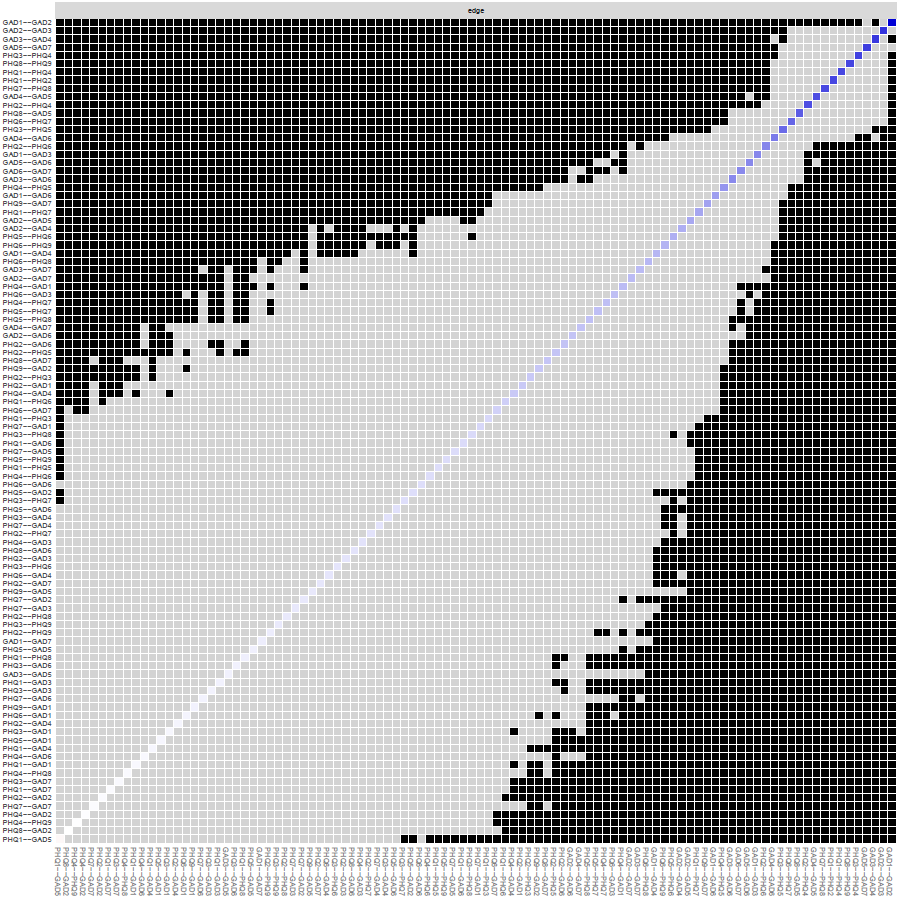


Figure S2. Bootstrapped stability test for edge-weight

PHQ1: Anhedonia, PHQ2: Sad Mood, PHQ3: Sleep, PHQ4: Fatigue, PHQ5: Appetite, PHQ6: Guilty, PHQ7: Concentration, PHQ8: Motor, PHQ9: Suicide; GAD1: Nervousness, GAD2: Uncontrollable worry, GAD3: Excessive worry, GAD4: Trouble relaxing, GAD5: Restlessness, GAD6: Irritability, GAD7: Feeling afraid; The results of the bootstrapped difference tests (α=0.05) for edge-weights were shown in this figure. There was a total of (16*(16-1))/2=120 edges between all 16 symptoms. The color of the boxes indicates whether edge-weights differ significantly from each other (i.e., black) or do not differ significantly (i.e., grey). The diagonal line indicates the strength of edge-weights, shifting from red (negative associations), to white (representing weaker edges) and ultimately blue (representing stronger edge-weights).


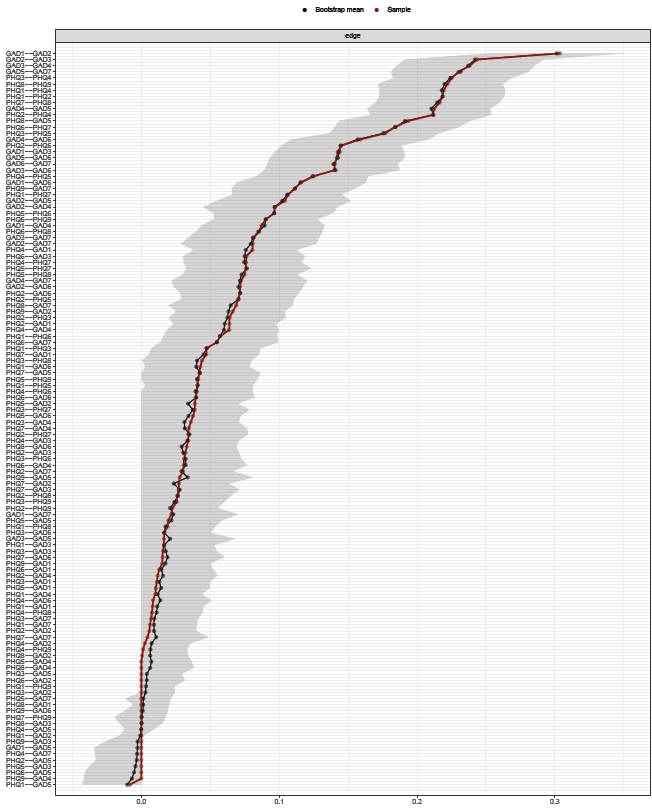


Figure S3. Bootstrapped confidence intervals of all edges

PHQ1: Anhedonia, PHQ2: Sad Mood, PHQ3: Sleep, PHQ4: Fatigue, PHQ5: Appetite, PHQ6: Guilty, PHQ7: Concentration, PHQ8: Motor, PHQ9: Suicide; GAD1: Nervousness, GAD2: Uncontrollable worry, GAD3: Excessive worry, GAD4: Trouble relaxing, GAD5: Restlessness, GAD6: Irritability, GAD7: Feeling afraid; The red dots indicate the values of each edge weight from the network and the black dots indicate the value of each edge weight by bootstrap procedure, both ordered from the highest to the lowest value. The gray area represents the 95% Confidence Intervals of edge weights, estimated with the non-parametric bootstrap procedure. Wide intervals indicate lower stability and narrow intervals indicate higher stability.


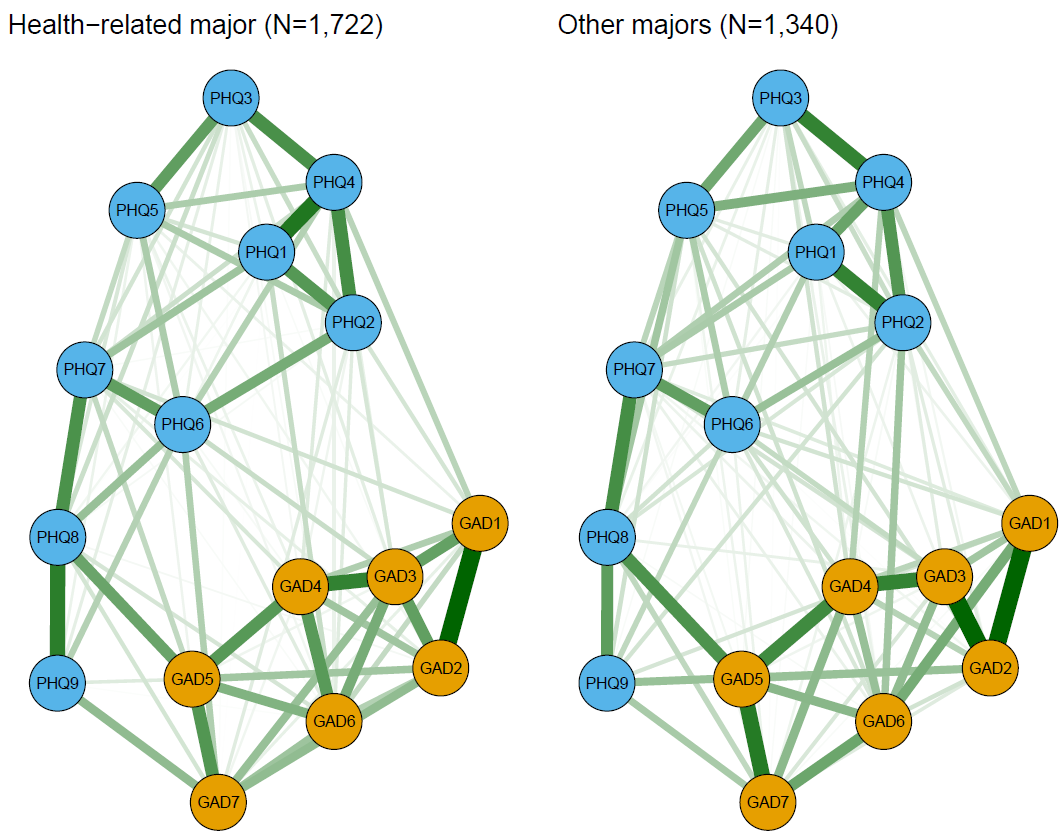


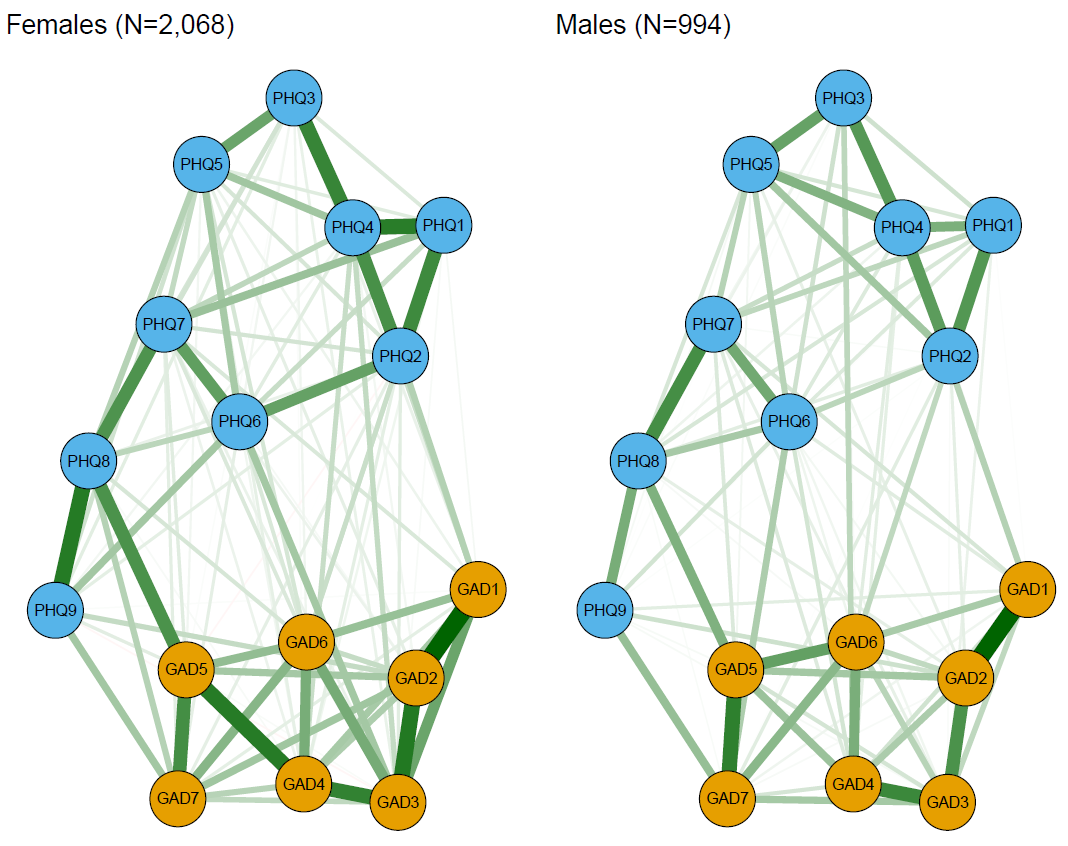


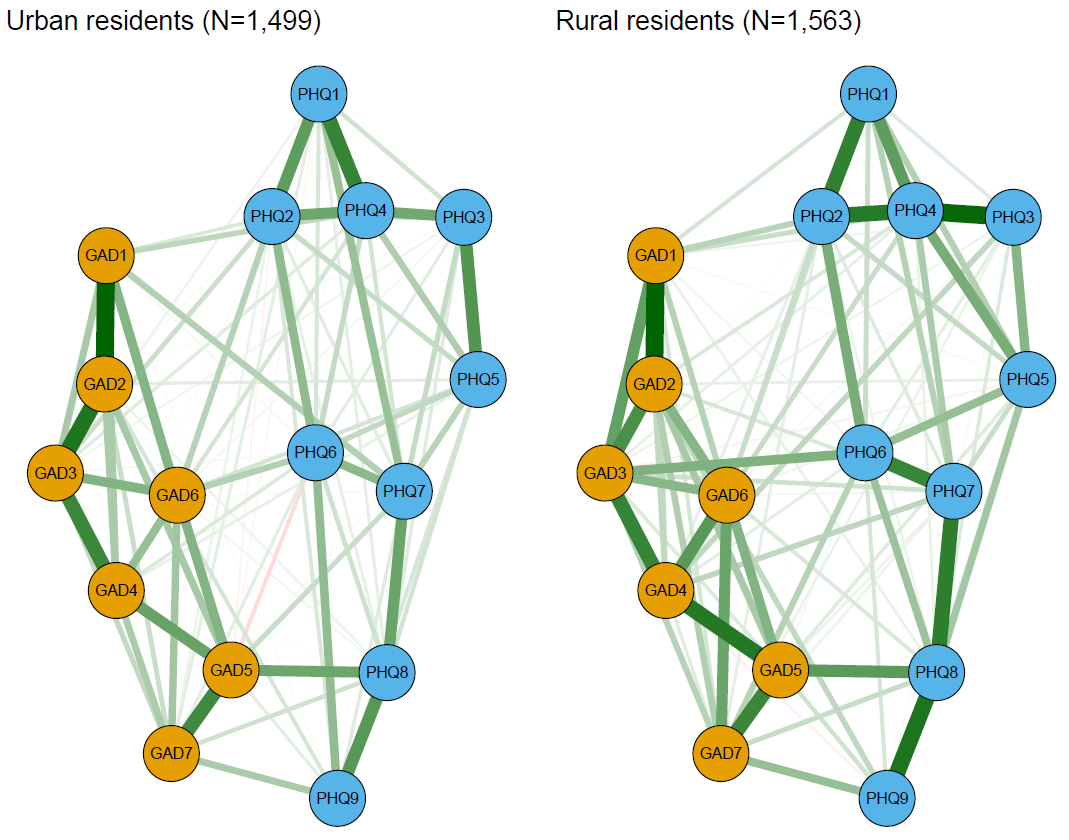
 Figure S4. Network comparisons of anxiety and depressive symptoms by academic majors, genders and living areas in college students

PHQ1: Anhedonia, PHQ2: Sad Mood, PHQ3: Sleep, PHQ4: Fatigue, PHQ5: Appetite, PHQ6: Guilty, PHQ7: Concentration, PHQ8: Motor, PHQ9: Suicide; GAD1: Nervousness, GAD2: Uncontrollable worry, GAD3: Excessive worry, GAD4: Trouble relaxing, GAD5: Restlessness, GAD6: Irritability, GAD7: Feeling afraid.
